# Medico-Surgical Aspects of the Spanish-American War

**Published:** 1901-05

**Authors:** 


### Books and Magazines.

---

MEDICO-SURGICAL ASPECTS OF THE SPANISH-AMERICAN WAR.—By Lieutenant-Colonel Dr. Nicholas Senn, Chief Surgeon United States Volunteers, Chief of Operating Staff with the Army in the Field, Professional Lecturer on Military Surgery, Chicago University. Published by the American Medical Association Press. 1900. Cloth; 380 pages.

This book is made up of a series of about twenty-seven articles, written by Lieutenant-Colonel Senn during the Spanish-American war. Most of these articles are interesting, and well worth reading. Especially is this true of those on "Military Surgery after the Battle of Santiago," "Typhoid Fever in the Porto Rican Campaign," and "Nurses and Nursing in War."

In appearance the book is attractive. It is neatly bound in cloth, well printed and fairly well illustrated. W. B. R.

